# Invited Letter to editor (respons STRE-D-24–00176)

**DOI:** 10.1186/s13049-024-01223-z

**Published:** 2024-06-07

**Authors:** Risco van Vliet, Lennert Breedveld, Annemieke A. J. Heutinck, Bram H. A. Ockeloen, Arnoud W. J. van ’t Hof, Xavier R. J. Moors

**Affiliations:** 1 Regional Emergency Medical Services, RAV Brabant Midden-West-Noord, ‘S-Hertogenbosch, the Netherlands; 2 Department of Cardiology, Maastricht University Medical Center, Maastricht, the Netherlands; 3 Department of Anesthesiology, Erasmus University Medical Center, Sophia Children’s Hospital, Rotterdam, the Netherlands; 4 Department of Cardiology, Zuyderland Medical Centre, Heerlen, the Netherlands; 5 Cardiovascular Research Instititute Maastricht (CARIM), Maastricht, the Netherlands

Thank you for the opportunity to comment on the "letter to the editor" written by Sarper Yilmaz, Assoc. Prof. Rohat Ak, Assoc. Prof. Ali Cankut Tatlıparmak, Assoc. Prof.

By having a public discussion, we gain a better understanding of each other's thoughts and hope that we can bring each other closer together on a professional level. Even though health systems are substantially different, it is ultimately about the safety of care for our prehospital patients. It is a challenge to thoroughly describe our specific prehospital field, with all its challenges and opportunities, so that it can be well understood from other perspectives. We regret that we have not managed to do this sufficiently, causing others to experience ambiguities. Therefore, we would like to take advantage of this invitation by answering the questions and concerns with the expectation of creating more understanding.

A concern is raised that the use of propofol could become part of standard pain management protocols. Propofol is a sedative, not an analgesic, and therefore has no place in our nationwide ambulance pain protocol. It is certainly not our ambition to add this as a standard to the protocol, nor do we want to promote it. Performing a “Procedural Sedation and Analgesia” (PSA) and applying sedation by using propofol is only allowed by advanced practice providers (APP) [1]. These APPs are qualified nurse practitioners or physician assistants working for the Dutch ambulance service “RAV Brabant MWN”. They are either postgraduate Master of Science-trained nurse practitioners or postgraduate Master of Science-trained physician assistants (Netherlands/European Qualifications Framework, Level 7). Before this APP is allowed to perform this procedure at all, it undergoes an intensive three-day PSA training course that is similar to that followed by, for example, emergency physicians, and which concludes with an exam. Components of this training include pharmacodynamics and pharmacokinetics, indication, performance of the procedure, and management of potential complications. In addition, this act is always performed in collaboration with an ambulance unit (advanced life support unit). All PSAs are supervised (by telephone) by an anesthesiologist or two emergency physicians.

There is concern about the risk of severe hypotension in these trauma patients. Despite that is indeed a potential danger, it is anticipated by applying the following agreements and actions.

## Selection of patients

Performing a PSA is done only in patients with a unilateral injury. For example, alignment of fractured extremity or repositioning of luxated joint, often caused by a non-high-energy accident. Pre-existing hazards such as high Body mass index (BMI) or ASA classification [2] are also sought in advance. So that the risk of pre-existing complications is low, which includes monitoring vital parameters. In the case of multi trauma, the risk of complications increases and support from a physician-staffed service (i.e. HEMS) is requested. In this manuscript all patients who underwent a PSA after trauma were not in hypovolemic shock.

## Choice of drug(s), dosing, and speed of administration

The national Dutch flow chart for analgesia allows the ambulance nurse to use paracetamol, fentanyl, and ketamine. However, the use of fentanyl and or ketamine causes side effects even in low doses such as vomiting, nausea, and dissociation. Especially in older frail elderly people. However, this standard of care with its side effects has never been adequately studied, nor is it known whether it affects the patient during his/her stay in the emergency room or hospital.

2–4 mg/kilogram is used for the introduction for anesthesia in normal healthy patients. The APP working for the Dutch EMS “RAV Brabant MWN” uses propofol in a low dose, not for induction but in a dosage for sedation. Propofol is administered slowly, starting with 0.5 mg/kilogram, and then titrated with a 5-10 mg bolus until the desired depth of sedation is reached. In addition, propofol has a short half-life and, unlike ketamine or fentanyl, it wears off quickly. To our regret, this was not described in the manuscript because we could not extract this data sufficiently and completely from the regular ambulance run sheets and database for the PSA. We now recognize that this would have been a valuable addition to the study limitations section.

## Preparation for complications

In preparation for any PSA or sedation, the APP takes several precautionary measures. For example, the patient is administered oxygen for preoxygenation and is given an IV containing 500 cc of Ringer lactate dripping to be able to give the medication a flush and have the potential for rapid infusion. The APP has emergency medication (adrenaline, atropine, ephedrine, or phenylephrine) at his/her disposal to deal with any complication of hypotension. The APP did not have to use this emergency medication during the interventions described in this manuscript from which it can be concluded that no complication of hypotension occurred.

We hope that by describing the above, we have given you more insight into the circumstances and preparation so that the APP can safely perform a PSA and/or apply sedation. Once again, I would like to emphasize that the differences in the organization of health systems and EMS services make it very difficult to compare systems with each other. To this end, we encourage more publications from the EMS services so that this important health care can be highlighted. Only then can we jointly evaluate and improve the quality of care.

## Data Availability

Not applicable.

## References

[1] van Vliet R, Breedveld L, Heutinck AAJ, Ockeloen BHA, van ’ Hof AWJ, Moors XRJ. Procedural sedation by advanced practice providers in the emergency medical service in the Netherlands: a retrospective study. Scand J Trauma Resusc Emerg Med [Internet]. 2024 Dec 1 [cited 2024 May 17];32(1). Available from: https://pubmed.ncbi.nlm.nih.gov/38693580/10.1186/s13049-024-01207-z PMC11064379 38693580

[2] Abouleish AE, Leib ML, Cohen NH (2015). ASA Provides Examples to Each ASA Physical Status Class. ASA Newsl.

